# Loose body following cross-pin fixation for anterior cruciate ligament reconstruction

**DOI:** 10.1007/s10195-012-0194-y

**Published:** 2012-04-17

**Authors:** R. A. Boden, A. Razak, S. M. A. Hussain, S. J. Mcloughlin

**Affiliations:** Orthopaedic Department, Blackpool Victoria Hospital, Whinney Heys Road, FY3 8NR Blackpool, UK

**Keywords:** Loose body, ACL reconstruction, RIGIDFIX, Bioabsorbable fixation

## Abstract

We report a case of loosening of a bioabsorbable cross-pin fixation device for anterior cruciate ligament reconstruction. Forty-two months following a bone tendon bone reconstruction of the anterior cruciate ligament, the patient presented with a subcutaneous collection in the medial side of the knee. At subsequent surgery, a RIGIDFIX cross-pin fixator (Mitek, Westwood, MA, USA) was retrieved, intact, from the sterile fluctuant mass around the superomedial aspect of the knee. The graft was stable both radiologically and clinically, and the patient remains symptom free. This case raises concern about the use of this smooth cross-pin fixator and the consequences of backing out and the resultant intraarticular loose body. We suggest consideration of a loose body if the patient becomes symptomatic postoperatively, and early intervention to prevent chondral damage is recommended.

## Introduction

Bioabsorbable implants are commonly used for orthopaedic intra-articular procedures. They have a theoretical advantage over metal analogues in their secure initial fixation and subsequent degradation with eventual replacement by host tissues [1]. The RIGIDFIX anterior cruciate ligament (ACL) cross pin (Mitek, Westwood, MA, USA) utilises polylactic (PLA) pins to give the advantage of 360° bone-to-graft contact at the graft tunnel, with clinically acceptable pull-out strength and proven patient outcomes [2, 3]. This fixation method does, however, have the disadvantages of local tissue response, such as inflammation and swelling [4, 5]. We report the case of implant migration and the loose body presenting as a subcutaneous collection.

## Case report

A 25-year-old fit and well male sustained a left complete ACL rupture in August 2001 due to a rotational soccer injury. This was confirmed at subsequent arthroscopy, and after a course of physiotherapy and a period of nonoperative treatment he elected for a reconstruction. This procedure was performed 3 years after the initial injury in August 2004 using bone patellar tendon bone (BTB) without complication. 9 and 10 mm bone plugs were harvested and fixed in the femoral canal with two RIGIDFIX cross pins placed from the lateral side of the femur as per the standard operative technique. The tibia was fixed with a 20 mm × 9 mm metal interference screw. A concomitant posterior 1/3 tear of the medial meniscus was repaired at the time of operation with 2 Mitek bioabsorbable sutures.

**Fig. 1:**
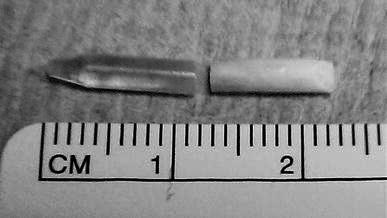
Retrieved RIGIDFIX cross pin

Postoperatively, the patient made a full recovery and returned to sporting activity at 6 months. Whilst on holiday in February 2008, the patient developed an acute collection on the medial side of his knee overlying the medial femoral condyle. This was incised and drained, and a 22 mm fragment consistent with the RIGIDFIX implant was retrieved. The fragment was retrieved intact and later fractured (Fig. 1). The wound healed and was treated with antibiotics, although no evidence of infection was documented. The patient’s inflammatory markers were normal and at last follow-up there was no evidence of superficial or deep infection. The range of movement in the knee was 0°–120° and the ACL was stable based on the normal anterior drawer and Lachman tests.

**Fig. 2:**
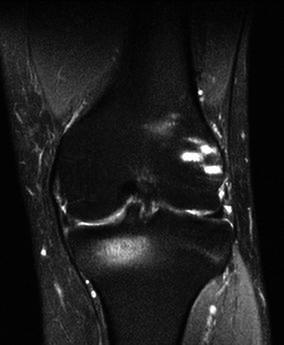
Post operative MRI scan showing intact ACL and healed medial meniscus (coronal view)

**Fig. 3:**
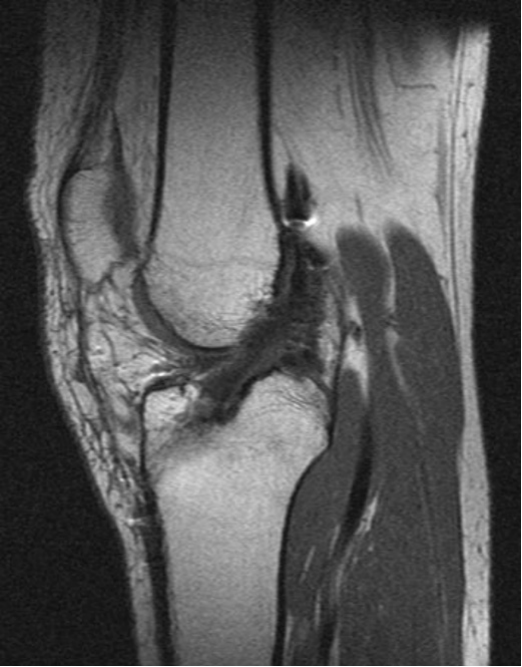
Post operative MRI scan showing intact ACL (sagittal view)

Magnetic resonance imaging (MRI) undertaken in October 2008 demonstrated an intact reconstructed ACL with a well-healed medial meniscus (Figs. 2, 3).

The patient gave informed consent prior to being included in the study.

## Discussion

We believe this to be the second reported case of loosening of the RIGIDFIX bioabsorbable pin for BTB ACL reconstruction [5]. Unlike in the previous case, where the entire pin was found in two sections within the joint, we describe a subcutaneous PLA implant fragment that caused local inflammation within the tissues on the medial aspect of the knee.

Cross-pin transverse femoral fixation in ACL reconstruction principally leads to increased pull-out strength of the graft. This, however, can lead to backing out of the pin if not positioned correctly, which is not the case in this patient, as the operation was done according to the standard RIGIDFIX surgical technique.

The timing of the backing out of the pin is unclear. What was apparent, however, was that the graft appeared to incorporate well on MRI and give a clinically acceptable outcome, even after losing one pin [3, 4].

The PLA implant remained unabsorbed 42 months postoperatively (Fig. 1). This is in keeping with previous studies demonstrating the presence of bioabsorbable implant remnants up to 5.7 years after implantation [1, 4]. While the backing out of these smooth pins did not cause any loss of ACL function in this case, it did result in the implant acting as a loose body. This loose body was in contact with the joint for an undefined period of time before it became embedded in the medial soft tissues.

This intraarticular contact theoretically placed the joint at risk of both mechanical and chemical damage [1]. Chemical damage results from the reduced pH associated with material breakdown. This is thought to promote inflammation, and has been directly linked to a reduction in cultured cell growth and possible chondral damage [6]. Local clearance by macrophages and polymophonuclear leukocytes is thought to be overloaded by excessive breakdown of bioabsorbable implants, which in theory could be increased by the presence of a loose PLA pin in the intra-articular space.

It is unclear as to the stage at which the pin backed out, and therefore how long the loose body was indeed loose. The fragment was also discovered in the subcutaneous tissues on the contralateral side of the knee to its insertion point, so it can only be postulated that it must have travelled through the joint itself.

While bioabsorbable implants do have the benefits of good initial fixation and a larger surface area for integration than interference screws, they do carry a risk of backing out and the subsequent loose body reaction. We have not been able to determine the cause of the pins backing out in this case. It is, however, a rare occurrence for what is a well-tolerated operation with good functional outcome [3]. It is worth considering a loose body from this graft in the event of knee pain or failure to progress in the postoperative period.
